# Supplementary material for: Pathogenicity Analyses of Rice Blast Fungus (Pyricularia oryzae) from Japonica Rice Area of Northeast China
Source: Pathogens. 2024 Feb 28;13(3):211. doi: 10.3390/pathogens13030211 (PMC10976127; doi:10.3390/pathogens13030211)
Supplement: Supplementary file 1 [file pathogens-13-00211-s001.zip › SUPPLEMENTARY Tables S1 and S2.pdf]

Supplementary Table S1. Total 206 rice blast races information according to U-i-k-z-ta pattern named criteria.

Code	Race Type	Amount	Isolate(s) site(s)	Code	Race Type	Amount	Isolate(s) site(s)
1	U01-i0-k000-z00-ta320	1	JL17	89	U72-i3-k177-z07-ta330	1	SY12
2	U01-i0-k020-z05-ta000	1	TH29	90	U72-i4-k102-z01-ta330	1	SP2
3	U03-i7-k177-z03-ta331	1	SY21	91	U72-i5-k103-z01-ta130	1	SY8
4	U11-i2-k000-z05-ta300	1	SY6	92	U72-i5-k117-z02-ta332	1	JL26
5	U13-i1-k171-z14-ta330	1	YJ8	93	U72-i5-k177-z01-ta330	1	YJ16
6	U13-i3-k143-z17-ta233	1	TH4	94	U72-i7-k137-z02-ta331	1	YJ2
7	U13-i5-k177-z07-ta330	1	CC28	95	U72-i7-k157-z03-ta332	1	SP3
8	U21-i1-k000-z00-ta330	1	TH8	96	U72-i7-k163-z03-ta330	1	CC20
9	U22-i4-k002-z00-ta030	1	JL22	97	U72-i7-k177-z15-ta330	1	SY23
10	U23-i1-k156-z01-ta230	1	YJ38	98	U72-i7-k177-z17-ta330	1	CC27
11	U23-i4-k000-z04-ta300	1	SY32	99	U73-i1-k001-z00-ta330	1	TH21
12	U23-i4-k112-z15-ta000	1	SY18	100	U73-i1-k003-z00-ta221	1	LY16
13	U23-i5-k173-z00-ta330	1	SP17	101	U73-i1-k010-z01-ta330	1	CC2
14	U31-i5-k147-z01-ta230	1	YJ30	102	U73-i1-k100-z01-ta330	1	SY11
15	U32-i2-k147-z11-ta330	1	YJ35	103	U73-i1-k100-z01-ta730	1	TH27
16	U33-i3-k157-z17-ta730	1	YJ33	104	U73-i1-k101-z00-ta230	1	JL16
17	U33-i3-k177-z14-ta730	1	YJ31	105	U73-i1-k101-z00-ta330	2	SY10, TH28
18	U33-i5-k177-z13-ta730	1	TH10	106	U73-i1-k101-z01-ta330	2	JL15, CC6
19	U33-i7-k177-z05-ta232	1	YJ32	107	U73-i1-k101-z01-ta332	2	LY24, TH23
20	U40-i5-k154-z01-ta320	1	YJ3	108	U73-i1-k101-z01-ta333	1	LY2
21	U43-i1-k167-z17-ta320	1	TH22	109	U73-i1-k101-z01-ta730	1	CC25
22	U43-i4-k111-z01-ta132	1	YJ4	110	U73-i1-k101-z03-ta330	1	JL29
23	U43-i4-k147-z11-ta320	1	YJ12	111	U73-i1-k101-z11-ta330	1	SP7
24	U52-i1-k003-z02-ta312	1	LY9	112	U73-i1-k101-z17-ta733	1	JL14
25	U52-i1-k100-z01-ta330	1	CC7	113	U73-i1-k102-z00-ta330	1	TH15
26	U52-i5-k114-z04-ta130	1	SP18	114	U73-i1-k103-z00-ta021	1	LY15
27	U52-i5-k177-z07-ta330	1	CC5	115	U73-i1-k111-z01-ta330	1	CC29
28	U52-i5-k177-z17-ta332	1	CC14	116	U73-i1-k143-z01-ta130	1	LY13
29	U52-i7-k177-z07-ta330	1	CC4	117	U73-i1-k177-z01-ta732	1	YJ29
30	U52-i7-k177-z07-ta731	1	SY13	118	U73-i1-k177-z17-ta733	1	CC1
31	U53-i1-k101-z01-ta330	1	JL21	119	U73-i2-k057-z15-ta330	1	YJ28
32	U53-i1-k101-z02-ta330	3	TH13, YJ18, YJ25	120	U73-i3-k103-z00-ta330	1	TH6
33	U53-i1-k101-z14-ta010	1	SY28	121	U73-i3-k123-z16-ta330	1	LY17
34	U53-i1-k177-z01-ta332	1	SP9	122	U73-i3-k145-z15-ta732	1	YJ11
35	U53-i3-k177-z15-ta330	1	LY18	123	U73-i3-k175-z03-ta330	1	YJ36
36	U53-i4-k131-z01-ta320	1	SY19	124	U73-i3-k177-z02-ta130	1	YJ26
37	U53-i5-k067-z03-ta330	1	YJ37	125	U73-i4-k102-z00-ta220	1	SP15
38	U53-i5-k131-z07-ta620	1	CC13	126	U73-i5-k177-z12-ta330	1	JL18
39	U53-i5-k157-z07-ta330	1	JL19	127	U73-i5-k032-z02-ta713	1	SY1
40	U53-i5-k165-z07-ta332	1	JL3	128	U73-i5-k055-z17-ta730	1	YJ34

41	U53-i5-k174-z01-ta330	1	YJ15	129	U73-i5-k101-z00-ta330	1	CC26
42	U53-i5-k175-z01-ta730	1	TH9	130	U73-i5-k101-z01-ta330	2	JL7, JL13
43	U53-i5-k175-z02-ta332	1	JL4	131	U73-i5-k101-z04-ta330	1	JL8
44	U53-i5-k175-z07-ta332	1	CC9	132	U73-i5-k102-z14-ta320	1	LY19
45	U53-i5-k175-z11-ta330	1	TH2	133	U73-i5-k103-z00-ta330	4	LY20, CC18, TH16, YJ19
46	U53-i5-k175-z17-ta332	1	SY31	134	U73-i5-k106-z04-ta730	1	SY30
47	U53-i5-k176-z01-ta332	1	SP19	135	U73-i5-k123-z00-ta130	1	TH5
48	U53-i5-k177-z01-ta031	1	TH12	136	U73-i5-k143-z00-ta330	1	SP14
49	U53-i5-k177-z10-ta330	1	SY3	137	U73-i5-k147-z15-ta330	1	SY16
50	U53-i5-k177-z17-ta331	1	SP8	138	U73-i5-k173-z01-ta731	1	SP16
51	U53-i5-k177-z17-ta733	1	SY7	139	U73-i5-k177-z01-ta330	1	JL11
52	U53-i6-k077-z17-ta230	1	LY7	140	U73-i5-k177-z01-ta730	2	YJ7, YJ22
53	U53-i6-k137-z06-ta330	1	CC12	141	U73-i5-k177-z03-ta332	1	TH17
54	U53-i7-k017-z17-ta331	1	JL30	142	U73-i5-k177-z07-ta330	3	SY2, JL10, LY10
55	U53-i7-k077-z17-ta731	1	YJ23	143	U73-i5-k177-z07-ta333	1	JL6
56	U53-i7-k175-z07-ta332	1	SP6	144	U73-i5-k177-z11-ta330	5	YJ20, JL28, LY21, YJ5, YJ27
57	U53-i7-k175-z07-ta730	2	SY27, SP1	145	U73-i5-k177-z11-ta332	2	JL25, YJ24
58	U53-i7-k175-z17-ta332	1	CC10	146	U73-i5-k177-z15-ta330	1	LY11
59	U53-i7-k177-z07-ta330	2	JL5, SP10	147	U73-i5-k177-z15-ta730	1	YJ40
60	U53-i7-k177-z07-ta332	1	SY17	148	U73-i5-k177-z15-ta731	1	JL20
61	U53-i7-k177-z07-ta730	1	CC21	149	U73-i6-k017-z01-ta330	1	JL2
62	U53-i7-k177-z07-ta732	1	LY22	150	U73-i7-k003-z00-ta330	1	SP20
63	U53-i7-k177-z13-ta731	1	TH14	151	U73-i7-k047-z05-ta330	1	YJ10
64	U53-i7-k177-z17-ta330	4	JL1, CC3, CC16, SP4	152	U73-i7-k145-z11-ta330	1	YJ13
65	U53-i7-k177-z17-ta730	1	JL24	153	U73-i7-k157-z01-ta730	1	YJ17
66	U53-i7-k177-z17-ta733	2	SY4, SP13	154	U73-i7-k165-z01-ta731	1	YJ39
67	U61-i0-k000-z00-ta000	1	LY12	155	U73-i7-k173-z07-ta332	1	TH19
68	U61-i5-k137-z17-ta732	1	CC24	156	U73-i7-k177-z01-ta730	1	LY14
69	U61-i5-k177-z17-ta330	1	LY3	157	U73-i7-k177-z01-ta732	1	SY14
70	U61-i7-k177-z17-ta330	1	CC31	158	U73-i7-k177-z03-ta330	2	JL9, CC11
71	U62-i3-k103-z01-ta000	1	SY9	159	U73-i7-k177-z03-ta332	1	SY20
72	U63-i1-k103-z04-ta330	1	CC19	160	U73-i7-k177-z07-ta330	1	TH26
73	U63-i1-k111-z01-ta330	1	TH25	161	U73-i7-k177-z07-ta332	1	LY5
74	U63-i5-k107-z01-ta322	1	SP5	162	U73-i7-k177-z07-ta333	1	TH3
75	U70-i1-k003-z00-ta330	1	LY8	163	U73-i7-k177-z07-ta731	1	SY29
76	U71-i0-k012-z10-ta220	1	SY24	164	U73-i7-k177-z07-ta733	1	SY15
77	U71-i1-k002-z00-ta330	1	TH7	165	U73-i7-k177-z13-ta120	1	YJ9
78	U71-i1-k102-z00-ta320	1	SY25	166	U73-i7-k177-z13-ta330	1	LY6
79	U71-i4-k157-z17-ta330	1	YJ21	167	U73-i7-k177-z15-ta330	1	YJ6
80	U71-i5-k177-z17-ta330	1	CC30	168	U73-i7-k177-z15-ta331	1	YJ14
81	U72-i0-k111-z01-ta120	1	TH18	169	U73-i7-k177-z15-ta730	1	TH1
82	U72-i1-k113-z03-ta333	1	CC22	170	U73-i7-k177-z17-ta332	1	CC17
83	U72-i1-k113-z07-ta332	1	CC8	171	U73-i7-k177-z17-ta333	2	SY22, SP11

84	U72-i1-k121-z01-ta330	1	JL12	172	U73-i7-k177-z17-ta431	1	LY23
85	U72-i1-k143-z02-ta330	1	CC23	173	U73-i7-k177-z17-ta731	3	CC15, TH11, TH24
86	U72-i1-k167-z07-ta230	1	TH20	174	U73-i7-k177-z17-ta732	2	LY1, SP12
87	U72-i3-k127-z01-ta330	1	YJ1	175	U73-i7-k177-z17-ta733	4	SY5, SY26, JL23, JL27
88	U72-i3-k143-z07-ta332	1	LY4	Total	--	206	--

Supplementary Table S2. Virulence differentials of rice blast isolates in many counties by MDVS.

Collected sample Site	high virulence	low virulence	moderate virulence
Cambodia	>60%, *Pit*, *Pia*, *Pi19*(t), *Pi20*(t), *Pib*, *Piz-t*	<20%, *Pish*, *Pik-m*, *Pi1*, *Pik-h*, *Pik*, *Pik-p*, *Pi7*(t), *Pi9*, *Piz*, *Pita-2* (two lines)	20-60%, *Pi3*, *Pii*, *Pi5*(t), *Pik-s*, *Piz-5*, *Pi12*(t), *Pita* (two lines)
Indonesia	>80%, *Pit*, *Pia*, *Pib*, *Pik-s*, *Pi12*(t)	<12.5%, *Pik-h*, *Pik*, *Pik-m*, *Pik-p*, *Pi1*, *Pi7*(t)	12.5-80%, *Pish*, *Pi3*, *Pii*, *Pi5*(t), *Pi19*(t), *Pi9*(t), *Pita-2* (two lines), *Piz-t*, *Piz-5*, *Pi20*(t), *Piz*, *Pita* (two lines)
Kenya	>50%, *Pia*, *Pii*, *Pik-s*, *Pi19*(t), *Pi20*(t), *Pib*, *Pi3*, *Pi5*(t), *Pi1*, *Pik-m*, *Pik-h*, *Pik*, *Pik-p*, *Pi7*(t)	<10%, *Pish*, *Pi9*(t), *Piz-t*, *Piz-5*	10-50%, *Piz*, *Pit*, *Pita*, *Pita-2*(two lines), *Pi12*(t)
Bangladesh	>60%, *Pit*, *Pia*, *Pii*, *Pik-s*, *Pi12*(t), *Pi19*(t), *Pi20*(t), *Pib*, *Piz-t*	<20%, *Pish*, *Pi9*, *Pita-2* (two lines), *Pita* (Ki)	20-60%, *Pi3*, *Pi5*(t), *Pi1*, *Pik-m*, *Pik-h*, *Pik*, *Pik-p*, *Pi7*(t), *Piz*, *Piz-5*, *Pita* (CP1)
Japan	>82.5%, *Pia*, *Pii*, *Pik-s*, *Pi19*(t), *Pi3*, *Pi5*(t), *Pish*	<21.6%, *Pi7*(t), *Piz-5*, *Pib*, *Piz-t*, *Piz*, *Pit*, *Pik-m*, *Pi12*(t), *Pi9*, *Pik*, *Pik-p*, *Pita-2* (two lines), *Pi1*, *Pik-h*, *Pi20*(t)	21.6-82.5%, *Pita*
Jilin province (China)	>80%: *Pish*, *Pit*, *Pia*, *Pii*, *Pik-s*, *Pik*, *Pita* (two lines) and *Pita-2* (two lines)	<40%: *Pi3*, *Pi9*(t), *Pi12*(t), *Pi19*(t) and *Pi20*(t)	40-80%: *Pib*, *Pi5*(t), *Pik-m*, *Pi1*, *Pik-h*, *Pik-p*, *Pi7*(t), *Piz*, *Piz-5* and *Piz-t*
