# Supplementary figures and images for: Pathogenicity Analyses of Rice Blast Fungus (Pyricularia oryzae) from Japonica Rice Area of Northeast China
Source: Pathogens. 2024 Feb 28;13(3):211. doi: 10.3390/pathogens13030211 (PMC10976127; doi:10.3390/pathogens13030211)

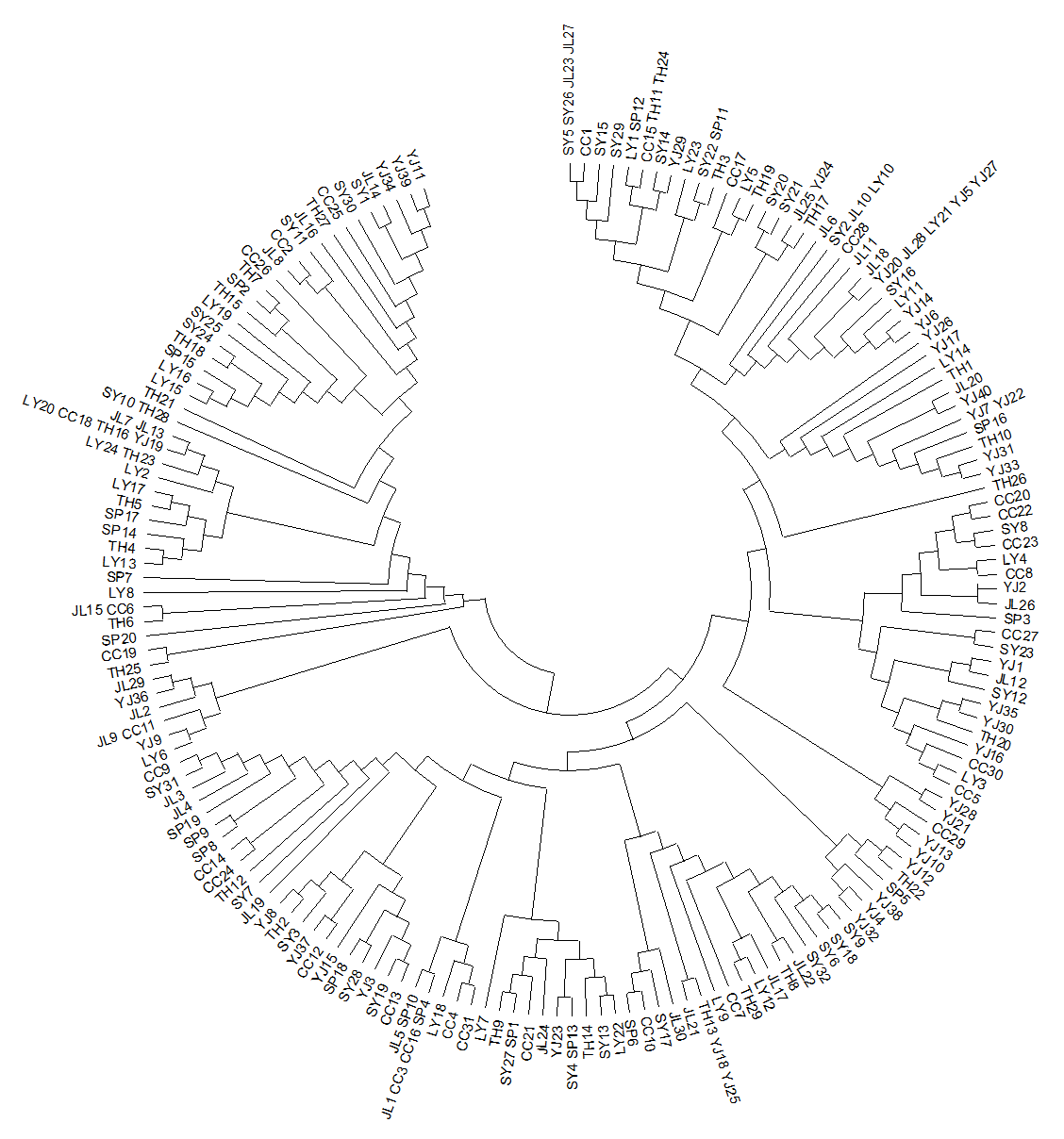

Supplement: Supplementary file 1 [file pathogens-13-00211-s001.zip › S1.png]
